# Bis(methanol-1κ*O*)tetra-μ-pyridazine-1:2κ^4^ *N*:*N*′;2:3κ^4^ *N*:*N*′-di-μ-thio­cyanato-1:2κ^2^ *N*:*N*;2:3κ^2^ *N*:*N*-tetrathio­cyanato-1κ^2^ *N*,3κ^2^ *N*-trinickel(II) methanol tetra­solvate

**DOI:** 10.1107/S1600536812026864

**Published:** 2012-06-16

**Authors:** Susanne Wöhlert, Inke Jess, Christian Näther

**Affiliations:** a Institut für Anorganische Chemie, Christian-Albrechts-Universität Kiel, Max-Eyth-Strasse 2, 24118 Kiel, Germany

## Abstract

Reaction of an excess nickel(II) thio­cyanate with pyridazine leads to single crystals of the title compound, [Ni_3_(NCS)_6_(N_2_C_4_H_4_)_4_(CH_3_OH)_2_]·4CH_3_OH. The crystal structure consists of trimeric discrete complexes, in which two Ni^II^ cations are coordinated by two terminal and one *μ*-1,1 bridging thio­cyanato anions, one methanol mol­ecule and two bridging pyridazine ligands, whereas the central Ni^II^ atom is coordinated by two *μ*-1,1 bridging anions as well as four bridging pyridazine ligands. The asymmetric unit consists of two crystallographically independent Ni cations, one of which is located on a center of inversion, as well as three crystallographically independent thio­cyanato anions, two pyridazine ligands and three independent methanol mol­ecules in general positions. Two of the solvent mol­ecules do not coordinate to the metal atoms and are located in cavities of the structure. The discrete complexes are linked by inter­molecular O—H⋯O and O—H⋯S hydrogen bonding into layers parallel to the *bc* plane.

## Related literature

For the background to this work and the synthesis of bridging thio­cyanato coordination compounds, see: Boeckmann & Näther (2010 ▶, 2011 ▶); Wöhlert *et al.* (2011 ▶). For structures of related trinuclear complexes, see: Wriedt & Näther (2009 ▶); Yi *et al.* (2006 ▶). For a description of the Cambridge Structural Database, see: Allen (2002 ▶). 
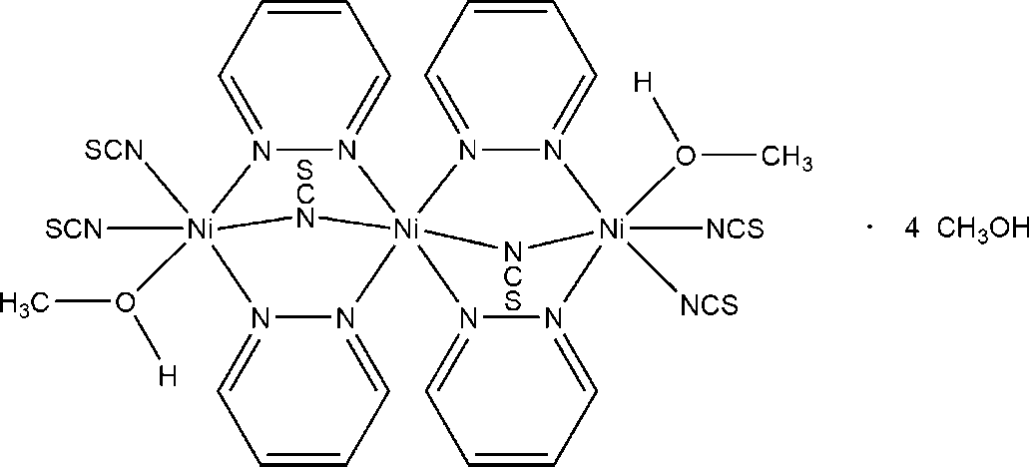


## Experimental

### 

#### Crystal data

[Ni_3_(NCS)_6_(C_4_H_4_N_2_)_4_(CH_4_O)_2_]·4CH_4_O
*M* *_r_* = 1037.23
Orthorhombic, 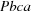

*a* = 17.6689 (12) Å
*b* = 15.0760 (7) Å
*c* = 17.9479 (10) Å
*V* = 4780.9 (5) Å^3^
*Z* = 4
Mo *K*α radiation
μ = 1.48 mm^−1^
*T* = 200 K
0.13 × 0.09 × 0.07 mm

#### Data collection

Stoe IPDS-1 diffractometer
Absorption correction: numerical (*X-SHAPE* and *X-RED32*; Stoe & Cie, 2008 ▶) *T*_min_ = 0.746, *T*_max_ = 0.818
31891 measured reflections
4093 independent reflections
3190 reflections with *I* > 2σ(*I*)
*R*_int_ = 0.065

#### Refinement

*R*[*F* ^2^ > 2σ(*F* ^2^)] = 0.041
*wR*(*F* ^2^) = 0.106
*S* = 1.05
4093 reflections
263 parameters
H-atom parameters constrained
Δρ_max_ = 0.34 e Å^−3^
Δρ_min_ = −0.52 e Å^−3^

### 

Data collection: *X-AREA* (Stoe & Cie, 2008 ▶); cell refinement: *X-AREA*; data reduction: *X-AREA*; program(s) used to solve structure: *SHELXS97* (Sheldrick, 2008 ▶); program(s) used to refine structure: *SHELXL97* (Sheldrick, 2008 ▶); molecular graphics: *XP* in *SHELXTL* (Sheldrick, 2008 ▶) and *DIAMOND* (Brandenburg, 2011) ▶; software used to prepare material for publication: *XCIF* in *SHELXTL*.

## Supplementary Material

Crystal structure: contains datablock(s) I, global. DOI: 10.1107/S1600536812026864/hp2040sup1.cif

Structure factors: contains datablock(s) I. DOI: 10.1107/S1600536812026864/hp2040Isup2.hkl

Additional supplementary materials:  crystallographic information; 3D view; checkCIF report

## Figures and Tables

**Table 1:** Selected bond lengths (Å)

Ni1—N3	2.024 (3)
Ni1—N1	2.031 (3)
Ni1—O1	2.067 (3)
Ni1—N10	2.110 (3)
Ni1—N20	2.128 (3)
Ni1—N2	2.132 (3)
Ni2—N21	2.099 (3)
Ni2—N2	2.114 (3)
Ni2—N11	2.121 (3)

**Table 2:** Hydrogen-bond geometry (Å, °)

*D*—H⋯*A*	*D*—H	H⋯*A*	*D*⋯*A*	*D*—H⋯*A*
O1—H1*O*1⋯O2^i^	0.84	1.84	2.671 (4)	171
O2—H1*O*2⋯O3	0.84	1.91	2.691 (9)	155
O3—H1*O*3⋯S1	0.84	2.45	3.285 (6)	178

Symmetry code: (i) 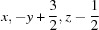.

**Fig. 1.**
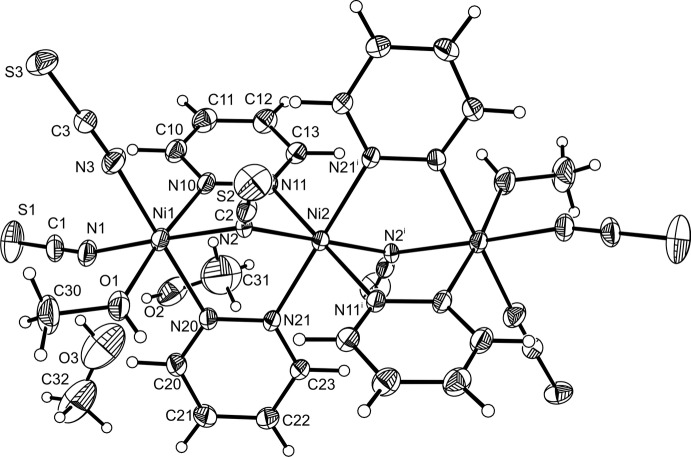
: Crystal structure of the title compound with labeling and displacement ellipsoids drawn at the 30% probability level. Symmetry code: i = -x + 1, -y + 1, -z + 1.

**Fig. 2.**
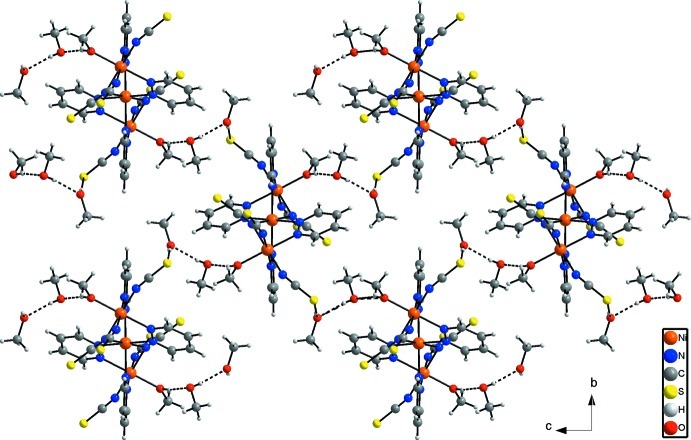
: Crystal structure of the title compound with view along the a-axis. Intermolecular O—H···O and O—H···S hydrogen bonding is shown as dashed lines.

## supplementary crystallographic information

### Comment

The structure determination of the title compound was performed within a project on the synthesis of transition metal coordination compounds in which the metal centers are linked by bridging anionic ligands (Boeckmann & Näther (2010, 2011); Wöhlert *et al.* (2011)). Within this project we reported on two modification of a trinuclear complex based on nickel(II) thiocyanate and pyridazine (Wriedt & Näther (2009)). In further investigations we have reacted nickel(II) thiocyanate with pyridazine in methanol which results in the formation of single-crystals of the title compound, which were characterized by single-crystal X-ray diffraction. The asymmetric unit of the title compound consists of two nickel(II) cations, one of them is located on a center of inversion, three thiocyanato anions, two pyridazine ligands and three methanol molecules all of them located in general position (Fig. 1). In the crystal structure two crystallographic independent nickel(II) cations are present. Ni1 is coordinated by two terminal N-bonded and one *µ*-1,1 bridging thiocyanato anions, one methanol molecule and two bridging pyridazine ligands in a slightly distorted octahedral geometry (Tab. 1). Ni2 is coordinated by two *µ*-1,1 bridging thiocyanato anions and four pyridazine ligands and the coordination environment can also be described as a sligthly distorted octahedron (Tab. 1). The nickel(II) cations are connected through *µ*-1,1 bridging thiocyanato anions and the two µ_2_-*N,N* pyridazine ligands into trimeric units. The Ni—N distances are in range of 2.025 (3) Å to 2.133 (3) Å with angles between 86.53 (12) ° to 180 ° (Tab. 1). The intramolecular Ni···Ni distances amount to 3.3349 (4) Å. The crystal structure contains additional methanol molecules located in cavities of the structure which are not coordinated to the metal cations. These methanol molecules are linked by intermolecular O—H···O and O—H···S hydrogen bonding to the metal complexes forming layers which are parallel to the b-c plane (Fig. 2 and Tab. 2). It must be noted that according to a search in the CCDC database (CONQUEST Ver. 1 12.2010) (Allen, 2002) a trinuclear complex with cobalt(II) thiocyanate and pyridazine was reported by Yi *et al.* (2006).

### Experimental

Nickel(II) thiocyanate (Ni(NCS)_2_) and pyridazine were obtained from Alfa Aesar. All chemicals were used without further purification. 0.5 mmol (87.0 mg) and 0.125 mmol (9.1 µ*L*) pyridazine were reacted in 0.5 ml methanol. Green single crystals of the title compound were obtained after two days.

### Refinement

All H atoms were located in difference map but were positioned with idealized geometry and were refined isotropic with *U*_iso_(H) = 1.2 *U*_eq_(C) (1.5 for methyl H atoms) using a riding model with C—H = 0.95 for aromatic and 0.98 Å for methyl H atoms. The O—H H atoms were located in difference map, their bond lengths set to ideal values of 0.84 Å and afterwards they were refined using a riding model with *U*_iso_(H) = 1.5 *U*_eq_(O).

### Crystal data

**Table:**

[Ni_3_(NCS)_6_(C_4_H_4_N_2_)_4_(CH_4_O)_2_]·4CH_4_O	*F*(000) = 2136
*M**_r_* = 1037.23	*D*_x_ = 1.441 Mg m^−^^3^
Orthorhombic, *P**b**c**a*	Mo *K*α radiation, λ = 0.71073 Å
Hall symbol: -P 2ac 2ab	Cell parameters from 31891 reflections
*a* = 17.6689 (12) Å	θ = 2.6–25.0°
*b* = 15.0760 (7) Å	µ = 1.48 mm^−^^1^
*c* = 17.9479 (10) Å	*T* = 200 K
*V* = 4780.9 (5) Å^3^	Block, green
*Z* = 4	0.13 × 0.09 × 0.07 mm

### Data collection

**Table:**

Stoe IPDS-1 diffractometer	4093 independent reflections
Radiation source: fine-focus sealed tube	3190 reflections with *I* > 2σ(*I*)
Graphite monochromator	*R*_int_ = 0.065
phi scan	θ_max_ = 25.0°, θ_min_ = 2.6°
Absorption correction: numerical (*X-SHAPE* and *X-RED32*; Stoe & Cie, 2008)	*h* = −21→21
*T*_min_ = 0.746, *T*_max_ = 0.818	*k* = −17→16
31891 measured reflections	*l* = −21→21

### Refinement

**Table:**

Refinement on *F*^2^	Secondary atom site location: difference Fourier map
Least-squares matrix: full	Hydrogen site location: inferred from neighbouring sites
*R*[*F*^2^ > 2σ(*F*^2^)] = 0.041	H-atom parameters constrained
*wR*(*F*^2^) = 0.106	*w* = 1/[σ^2^(*F*_o_^2^) + (0.058*P*)^2^ + 2.6878*P*] where *P* = (*F*_o_^2^ + 2*F*_c_^2^)/3
*S* = 1.05	(Δ/σ)_max_ < 0.001
4093 reflections	Δρ_max_ = 0.34 e Å^−^^3^
263 parameters	Δρ_min_ = −0.52 e Å^−^^3^
0 restraints	Extinction correction: *SHELXL97* (Sheldrick, 2008), Fc^*^=kFc[1+0.001xFc^2^λ^3^/sin(2θ)]^-1/4^
Primary atom site location: structure-invariant direct methods	Extinction coefficient: 0.0022 (4)

### Special details

**Table:**

Geometry. All e.s.d.'s (except the e.s.d. in the dihedral angle between two l.s. planes) are estimated using the full covariance matrix. The cell e.s.d.'s are taken into account individually in the estimation of e.s.d.'s in distances, angles and torsion angles; correlations between e.s.d.'s in cell parameters are only used when they are defined by crystal symmetry. An approximate (isotropic) treatment of cell e.s.d.'s is used for estimating e.s.d.'s involving l.s. planes.
Refinement. Refinement of *F*^2^ against ALL reflections. The weighted *R*-factor *wR* and goodness of fit *S* are based on *F*^2^, conventional *R*-factors *R* are based on *F*, with *F* set to zero for negative *F*^2^. The threshold expression of *F*^2^ > σ(*F*^2^) is used only for calculating *R*-factors(gt) *etc*. and is not relevant to the choice of reflections for refinement. *R*-factors based on *F*^2^ are statistically about twice as large as those based on *F*, and *R*- factors based on ALL data will be even larger.

### Fractional atomic coordinates and isotropic or equivalent isotropic displacement parameters (Å^2^)

**Table:**

	*x*	*y*	*z*	*U*_iso_*/*U*_eq_	
Ni1	0.34256 (2)	0.62044 (3)	0.48362 (3)	0.03445 (16)	
Ni2	0.5000	0.5000	0.5000	0.02852 (17)	
N1	0.28878 (17)	0.7219 (2)	0.5360 (2)	0.0472 (8)	
C1	0.2580 (2)	0.7648 (3)	0.5794 (2)	0.0458 (9)	
S1	0.21556 (8)	0.82484 (12)	0.64235 (8)	0.0843 (5)	
N2	0.40392 (14)	0.51597 (19)	0.43107 (17)	0.0323 (6)	
C2	0.38567 (18)	0.4742 (3)	0.3823 (2)	0.0404 (9)	
S2	0.36077 (8)	0.41172 (10)	0.31352 (7)	0.0706 (4)	
N3	0.24364 (17)	0.5553 (2)	0.46794 (19)	0.0472 (8)	
C3	0.1898 (2)	0.5208 (3)	0.44548 (19)	0.0376 (8)	
S3	0.11289 (6)	0.47346 (8)	0.41480 (6)	0.0539 (3)	
N10	0.36318 (14)	0.5577 (2)	0.58671 (16)	0.0340 (7)	
N11	0.42715 (14)	0.50917 (19)	0.59358 (16)	0.0322 (6)	
C10	0.3176 (2)	0.5655 (3)	0.6453 (2)	0.0434 (9)	
H10	0.2721	0.5982	0.6397	0.052*	
C11	0.3333 (2)	0.5282 (3)	0.7143 (2)	0.0500 (10)	
H11	0.3003	0.5367	0.7556	0.060*	
C12	0.3977 (2)	0.4789 (3)	0.7208 (2)	0.0471 (10)	
H12	0.4109	0.4513	0.7666	0.057*	
C13	0.4432 (2)	0.4704 (3)	0.65802 (19)	0.0381 (8)	
H13	0.4878	0.4354	0.6614	0.046*	
N20	0.44811 (15)	0.6868 (2)	0.49603 (16)	0.0351 (6)	
N21	0.51239 (15)	0.63848 (19)	0.49894 (15)	0.0319 (6)	
C20	0.4517 (2)	0.7741 (3)	0.4989 (2)	0.0448 (9)	
H20	0.4059	0.8070	0.4978	0.054*	
C21	0.5200 (2)	0.8203 (3)	0.5035 (2)	0.0516 (10)	
H21	0.5210	0.8833	0.5049	0.062*	
C22	0.5851 (2)	0.7719 (3)	0.5060 (2)	0.0459 (9)	
H22	0.6331	0.7998	0.5096	0.055*	
C23	0.57830 (18)	0.6801 (2)	0.50301 (19)	0.0361 (8)	
H23	0.6232	0.6454	0.5039	0.043*	
O1	0.33376 (15)	0.6840 (2)	0.38195 (16)	0.0551 (8)	
H1O1	0.3691	0.6885	0.3509	0.083*	
C30	0.2715 (3)	0.7345 (4)	0.3553 (3)	0.0757 (16)	
H30A	0.2612	0.7833	0.3899	0.114*	
H30B	0.2268	0.6964	0.3516	0.114*	
H30C	0.2837	0.7586	0.3060	0.114*	
O2	0.4433 (2)	0.8203 (3)	0.7794 (2)	0.0830 (12)	
H1O2	0.4144	0.8368	0.7449	0.125*	
C31	0.4798 (6)	0.7414 (7)	0.7589 (6)	0.175 (5)	
H31A	0.5099	0.7515	0.7139	0.263*	
H31B	0.4419	0.6953	0.7492	0.263*	
H31C	0.5131	0.7221	0.7995	0.263*	
O3	0.3907 (4)	0.8965 (6)	0.6541 (4)	0.155 (3)	
H1O3	0.3461	0.8779	0.6501	0.232*	
C32	0.4027 (5)	0.9850 (8)	0.6309 (5)	0.139 (4)	
H32A	0.3727	1.0251	0.6622	0.209*	
H32B	0.3871	0.9915	0.5788	0.209*	
H32C	0.4564	0.9998	0.6358	0.209*	

### Atomic displacement parameters (Å^2^)

**Table:**

	*U*^11^	*U*^22^	*U*^33^	*U*^12^	*U*^13^	*U*^23^
Ni1	0.0228 (2)	0.0333 (3)	0.0473 (3)	0.00354 (17)	−0.00013 (17)	−0.00294 (19)
Ni2	0.0208 (3)	0.0264 (3)	0.0384 (3)	0.0023 (2)	0.0014 (2)	−0.0010 (2)
N1	0.0294 (15)	0.042 (2)	0.070 (2)	0.0081 (14)	−0.0004 (15)	−0.0047 (16)
C1	0.0334 (18)	0.041 (3)	0.063 (2)	0.0136 (17)	−0.0067 (17)	−0.0058 (19)
S1	0.0679 (8)	0.1127 (13)	0.0722 (8)	0.0446 (8)	−0.0053 (6)	−0.0333 (8)
N2	0.0231 (13)	0.0239 (16)	0.0501 (18)	0.0047 (11)	0.0058 (12)	0.0018 (13)
C2	0.0242 (17)	0.046 (2)	0.050 (2)	0.0069 (15)	0.0033 (15)	0.0134 (19)
S2	0.0730 (8)	0.0795 (10)	0.0592 (7)	−0.0096 (7)	−0.0096 (6)	−0.0177 (6)
N3	0.0301 (16)	0.051 (2)	0.060 (2)	−0.0013 (14)	−0.0017 (14)	−0.0052 (16)
C3	0.0341 (18)	0.036 (2)	0.0425 (19)	0.0008 (16)	0.0020 (15)	0.0024 (15)
S3	0.0455 (6)	0.0616 (8)	0.0546 (6)	−0.0195 (5)	−0.0071 (4)	0.0018 (5)
N10	0.0232 (13)	0.0337 (18)	0.0451 (16)	0.0009 (11)	0.0038 (11)	−0.0057 (12)
N11	0.0244 (13)	0.0290 (17)	0.0430 (15)	0.0017 (11)	0.0010 (11)	−0.0024 (12)
C10	0.0339 (18)	0.046 (2)	0.051 (2)	0.0026 (16)	0.0093 (16)	−0.0062 (17)
C11	0.047 (2)	0.054 (3)	0.049 (2)	−0.0013 (19)	0.0137 (17)	−0.0095 (18)
C12	0.046 (2)	0.057 (3)	0.0386 (19)	−0.0019 (19)	0.0024 (16)	−0.0018 (17)
C13	0.0321 (17)	0.039 (2)	0.0428 (19)	0.0007 (15)	0.0031 (15)	0.0005 (15)
N20	0.0281 (14)	0.0290 (18)	0.0481 (17)	0.0037 (12)	−0.0010 (12)	−0.0002 (12)
N21	0.0214 (13)	0.0326 (16)	0.0415 (15)	0.0031 (11)	0.0010 (11)	0.0007 (11)
C20	0.0324 (18)	0.030 (2)	0.072 (3)	0.0039 (14)	0.0003 (17)	0.0004 (18)
C21	0.046 (2)	0.030 (2)	0.079 (3)	−0.0041 (17)	−0.003 (2)	0.0016 (18)
C22	0.0332 (18)	0.037 (2)	0.067 (3)	−0.0069 (15)	−0.0010 (17)	0.0002 (18)
C23	0.0241 (16)	0.034 (2)	0.050 (2)	−0.0011 (13)	0.0007 (14)	0.0031 (15)
O1	0.0385 (15)	0.065 (2)	0.0616 (17)	0.0151 (13)	0.0002 (12)	0.0150 (14)
C30	0.052 (3)	0.101 (5)	0.074 (3)	0.032 (3)	−0.011 (2)	0.019 (3)
O2	0.063 (2)	0.092 (3)	0.094 (3)	−0.019 (2)	0.0164 (19)	−0.035 (2)
C31	0.180 (10)	0.115 (8)	0.231 (12)	0.009 (7)	0.093 (9)	−0.047 (8)
O3	0.107 (4)	0.235 (9)	0.122 (5)	−0.031 (5)	−0.010 (4)	−0.054 (5)
C32	0.080 (5)	0.248 (13)	0.090 (5)	−0.007 (7)	0.005 (4)	−0.025 (7)

### Geometric parameters (Å, º)

**Table:**

Ni1—N3	2.024 (3)	C13—H13	0.9500
Ni1—N1	2.031 (3)	N20—C20	1.319 (5)
Ni1—O1	2.067 (3)	N20—N21	1.350 (4)
Ni1—N10	2.110 (3)	N21—C23	1.325 (4)
Ni1—N20	2.128 (3)	C20—C21	1.395 (6)
Ni1—N2	2.132 (3)	C20—H20	0.9500
Ni2—N21^i^	2.099 (3)	C21—C22	1.363 (6)
Ni2—N21	2.099 (3)	C21—H21	0.9500
Ni2—N2	2.114 (3)	C22—C23	1.391 (5)
Ni2—N2^i^	2.114 (3)	C22—H22	0.9500
Ni2—N11^i^	2.121 (3)	C23—H23	0.9500
Ni2—N11	2.121 (3)	O1—C30	1.420 (5)
N1—C1	1.149 (5)	O1—H1O1	0.8399
C1—S1	1.631 (4)	C30—H30A	0.9800
N2—C2	1.126 (5)	C30—H30B	0.9800
C2—S2	1.614 (5)	C30—H30C	0.9800
N3—C3	1.156 (5)	O2—C31	1.403 (9)
C3—S3	1.631 (4)	O2—H1O2	0.8399
N10—C10	1.329 (5)	C31—H31A	0.9800
N10—N11	1.352 (4)	C31—H31B	0.9800
N11—C13	1.327 (5)	C31—H31C	0.9800
C10—C11	1.388 (6)	O3—C32	1.413 (11)
C10—H10	0.9500	O3—H1O3	0.8401
C11—C12	1.364 (6)	C32—H32A	0.9800
C11—H11	0.9500	C32—H32B	0.9800
C12—C13	1.391 (5)	C32—H32C	0.9800
C12—H12	0.9500		
			
N3—Ni1—N1	91.48 (13)	C10—C11—H11	121.2
N3—Ni1—O1	92.14 (13)	C11—C12—C13	117.6 (4)
N1—Ni1—O1	91.36 (13)	C11—C12—H12	121.2
N3—Ni1—N10	93.07 (13)	C13—C12—H12	121.2
N1—Ni1—N10	90.74 (13)	N11—C13—C12	122.8 (3)
O1—Ni1—N10	174.33 (10)	N11—C13—H13	118.6
N3—Ni1—N20	177.73 (13)	C12—C13—H13	118.6
N1—Ni1—N20	90.42 (12)	C20—N20—N21	119.8 (3)
O1—Ni1—N20	86.57 (11)	C20—N20—Ni1	121.1 (2)
N10—Ni1—N20	88.15 (11)	N21—N20—Ni1	119.2 (2)
N3—Ni1—N2	91.10 (12)	C23—N21—N20	119.1 (3)
N1—Ni1—N2	177.27 (12)	C23—N21—Ni2	124.2 (2)
O1—Ni1—N2	89.45 (11)	N20—N21—Ni2	116.7 (2)
N10—Ni1—N2	88.22 (11)	N20—C20—C21	122.9 (3)
N20—Ni1—N2	87.02 (11)	N20—C20—H20	118.6
N21^i^—Ni2—N21	180.0	C21—C20—H20	118.6
N21^i^—Ni2—N2	91.99 (11)	C22—C21—C20	117.6 (4)
N21—Ni2—N2	88.01 (11)	C22—C21—H21	121.2
N21^i^—Ni2—N2^i^	88.01 (11)	C20—C21—H21	121.2
N21—Ni2—N2^i^	91.99 (11)	C21—C22—C23	117.3 (3)
N2—Ni2—N2^i^	180.0	C21—C22—H22	121.3
N21^i^—Ni2—N11^i^	90.32 (10)	C23—C22—H22	121.3
N21—Ni2—N11^i^	89.68 (10)	N21—C23—C22	123.3 (3)
N2—Ni2—N11^i^	91.80 (11)	N21—C23—H23	118.4
N2^i^—Ni2—N11^i^	88.20 (11)	C22—C23—H23	118.4
N21^i^—Ni2—N11	89.68 (10)	C30—O1—Ni1	127.1 (3)
N21—Ni2—N11	90.32 (10)	C30—O1—H1O1	108.0
N2—Ni2—N11	88.20 (11)	Ni1—O1—H1O1	124.6
N2^i^—Ni2—N11	91.80 (11)	O1—C30—H30A	109.5
N11^i^—Ni2—N11	180.000 (1)	O1—C30—H30B	109.5
C1—N1—Ni1	163.4 (4)	H30A—C30—H30B	109.5
N1—C1—S1	178.9 (4)	O1—C30—H30C	109.5
C2—N2—Ni2	128.4 (3)	H30A—C30—H30C	109.5
C2—N2—Ni1	127.7 (3)	H30B—C30—H30C	109.5
Ni2—N2—Ni1	103.51 (13)	C31—O2—H1O2	109.7
N2—C2—S2	178.2 (4)	O2—C31—H31A	109.5
C3—N3—Ni1	167.6 (3)	O2—C31—H31B	109.5
N3—C3—S3	178.8 (4)	H31A—C31—H31B	109.5
C10—N10—N11	118.8 (3)	O2—C31—H31C	109.5
C10—N10—Ni1	123.4 (2)	H31A—C31—H31C	109.5
N11—N10—Ni1	117.8 (2)	H31B—C31—H31C	109.5
C13—N11—N10	119.8 (3)	C32—O3—H1O3	115.6
C13—N11—Ni2	122.1 (2)	O3—C32—H32A	109.5
N10—N11—Ni2	118.1 (2)	O3—C32—H32B	109.5
N10—C10—C11	123.4 (4)	H32A—C32—H32B	109.5
N10—C10—H10	118.3	O3—C32—H32C	109.5
C11—C10—H10	118.3	H32A—C32—H32C	109.5
C12—C11—C10	117.6 (3)	H32B—C32—H32C	109.5
C12—C11—H11	121.2		

Symmetry code: (i) −*x*+1, −*y*+1, −*z*+1.

### Hydrogen-bond geometry (Å, º)

**Table:**

*D*—H···*A*	*D*—H	H···*A*	*D*···*A*	*D*—H···*A*
O1—H1*O*1···O2^ii^	0.84	1.84	2.671 (4)	171
O2—H1*O*2···O3	0.84	1.91	2.691 (9)	155
O3—H1*O*3···S1	0.84	2.45	3.285 (6)	178

Symmetry code: (ii) *x*, −*y*+3/2, *z*−1/2.
